# Prevalence of Risk for Orthorexia in Athletes Using the ORTO-15 Questionnaire: A Systematic Mini-Review

**DOI:** 10.3389/fpsyg.2022.856185

**Published:** 2022-05-12

**Authors:** Ana Carolina Paludo, Marina Magatão, Hilana Rickli Fiuza Martins, Marcos Vinícius Soares Martins, Michal Kumstát

**Affiliations:** ^1^Incubator of Kinanthropology Research, Faculty of Sport Studies, Masaryk University, Brno, Czechia; ^2^Department of Physical Education, Universidade Estadual do Centro-Oeste, Guarapuava, Brazil; ^3^Department of Physical Therapy, Universidade Estadual do Centro-Oeste, Guarapuava, Brazil; ^4^Department of Physical Therapy, UniGuairacá, Guarapuava, Brazil; ^5^Department of Health Promotion, Faculty of Sport Studies, Masaryk University, Brno, Czechia

**Keywords:** athletes, eating disorders, food behavior, orthorexia nervosa, ORTO-15

## Abstract

The article aims to summarize the literature about the profile of risk of orthorexia in athletes using the ORTO-15 questionnaire. The search was performed at PubMed, Embase, Web of Science, and Sport Discus databases, using the terms “orthorexia” AND “athletes” with the respective entry terms. A multistage process of selection followed the PRISMA 2020 recommendation. A total of 688 articles were identified, and six studies were available for the final process. The prevalence of risk for orthorexia was assessed by the articles by the ORTO-15 questionnaire and ranged between 38 and 35 points. The comparison between male and female athletes and, athletes and non-athletes was not significant in the six articles. In conclusion, the review highlights that athletes from different sports, included in the review, do not present a risk of orthorexia nervosa considering the cutoff of 40 points, but not 35 points. Also, athletes present the same orthorexic behavior compared to non-athletes, demonstrating that orthorexia is an issue that needs to be considered in the general population. Moreover, a special focus should be given on the ORTO-15 questionnaire, about the sensitivity to diagnose the prevalence of orthorexia, especially in athletes.

## Introduction

The orthorexia nervosa (ON) introduces a behavioral pattern that has been classified as a pathological fixation on healthy nutrition. In this case, the individual with ON, also called orthorexic, is extremely concerned about the quality and quantity of food ingested in the diet. This specification with food quality can lead to a restrictive diet, leading to nutritional deficiencies, malnourishment, medical complications, and mental disorders (Koven and Abry, 2015). The ON is a new concept of disorder, introduced in the 1990s, that is not recognized as an eating disorder according to the Diagnostic and Statistical Manual of Mental Disorders (DSM-5) nor the International Statistical Classification of Diseases and Related Health Problems (ICD-10; Moroze et al., 2015; Dunn and Bratman, 2016), Definition of ON is in its earliest (Dunn and Bratman, 2016; Cena et al., 2019); however, there are several diagnostic tools available (Niedzielski and Kaźmierczak-Wojtaś, 2021).

The diagnosis of risk of orthorexia nervosa is mostly derived from a questionnaire of eating habits, in which Donini et al. (2005) developed the ORTO-15 questionnaire with data from the Italian population. This toll for the diagnosis of orthorexia includes 15 multiple-choice items that investigate the obsessive attitude of the subjects in choosing, buying, preparing, and consuming food considered healthy. Nowadays, the questionnaire is one of the most commonly psychometric tools used in studies to identify ON (Cena et al., 2019) and has been translated and validated in several languages (Pontes et al., 2014; Varga et al., 2014; Missbach et al., 2015; Stochel et al., 2015). To interpret the results from the questionnaire, a cutoff score set of 40 (with a score below 40) was able to correctly identify subjects believed to have ON; however, the authors point out that the cutoff point values can be set depending on the purpose for which the scales used (Donini et al., 2005).

Orthorexic behavior has been positively associated with perfectionism self-presentation (Pratt et al., 2022) or exercise addiction (Rudolph, 2018). Moreover, it has been demonstrated in people obsessed with a healthy lifestyle such as yoga practitioners (Herranz Valera et al., 2014; Domingues and Carmo, 2021), fitness sports (Rudolph, 2018), and users of social media (Turner and Lefevre, 2017); nonetheless, in sports settings, this relationship is still not summarized. In the context of athletic settings, disordered eating is a general term used to describe the spectrum of abnormal or harmful eating behaviors primarily used in an attempt to lose or maintain normal body weight to optimize performance. Lower body weight has a beneficial effect on athletic performance in some sports disciplines (e.g., endurance-based or aesthetic). To achieve desired weight outcomes, athletes often go on diets resulting in low energy availability, where the body is not adequately fueled, negatively affecting physiological functions needed for optimal health (Logue et al., 2018). This behavior attributes to the concept of Relative Energy Deficiency in Sport (RED-S) that IOC introduced recently and addressed serious health issues in both male and female athletes, beyond what is known as the female athlete triad (Reardon et al., 2019).

Therefore, the purpose of the present mini-review was to summarize the prevalence of risk for orthorexia nervosa in athletes, using the ORTO-15 questionnaire. In this review, we focus on competitive sports and with the specific use of the questionnaire ORTO-15 developed for this purpose. Moreover, it was also described the risk of orthorexia considering the cutoff of 40 and 35 points, as suggested elsewhere (Donini et al., 2005; Ramacciotti et al., 2011; Dell’Osso et al., 2016).

## Materials and Methods

The systematic mini-review was performed in accordance with the guidelines to the Preferred Reporting Items for Systematic Reviews and Meta-Analyses (PRISMA) updated in 2020 (Page et al., 2021), and the protocol was registered at PROSPERO with the number CRD42021288935.

### Eligibility Criteria for Selecting Studies

Studies were eligible for inclusion following the PECO criteria: Participants/Population (P): athletes, both sexes, of all sport disciplines. Exposure (E): studies that include data of orthorexia from athletes engaged in sports training programs. Comparator/control (C): The studies comparing data of orthorexia in professional athletes to non-athletes and/or male and female athletes. Outcomes (O) measures: prevalence of risk for orthorexia and cutoff point of ORTO-15. Non-English language articles, reviews or guidelines, conference abstracts, and dissertation thesis were excluded. Also, articles that not present a cutoff point for the risk of orthorexia were excluded. Only articles published until November 2021 were included.

### Search Strategy and Selection Process

A search strategy was performed on PubMed, Embase, Web of Science, and Sport Discus (*via* EBSCO*host*) databases, during November 2021. The search terms used PECO criteria, and a full search of each database was performed as follows: (orthorexia OR “orthorexia nervosa”) AND (“sport” OR “athlete” OR “professional athletes” OR “elite athletes” OR “elite athlete”). The data were imported into the Rayyan systematic review software to proceed with the selection process. A multistage process was performed, as follows: (i) one reviewer (AP) was used to include the articles that appeared in the search strategy in each database, (ii) after, the same reviewer excluded the repeated articles, then articles with review approach and no-English language, (iii) two independent reviewers (AP, MM) screening the title and abstract and one reviewer was nominated in case of disagreement (HM); (iv) two independent reviewers (AP and MM) screening the full text and one reviewer checked all studies excluded in this phase (HM).

### Data Collection Process

The extraction data included the sample characteristics (e.g., sample size, sport modality, sex, and age), risk of orthorexia (e.g., considering the cutoff of 40 points), comparison between groups (e.g., athlete versus non-athlete; and male versus female athletes), which was performed also for two researchers independently (AP and MM).

## Results

### Included Studies and Characteristics

**Figure 1:**
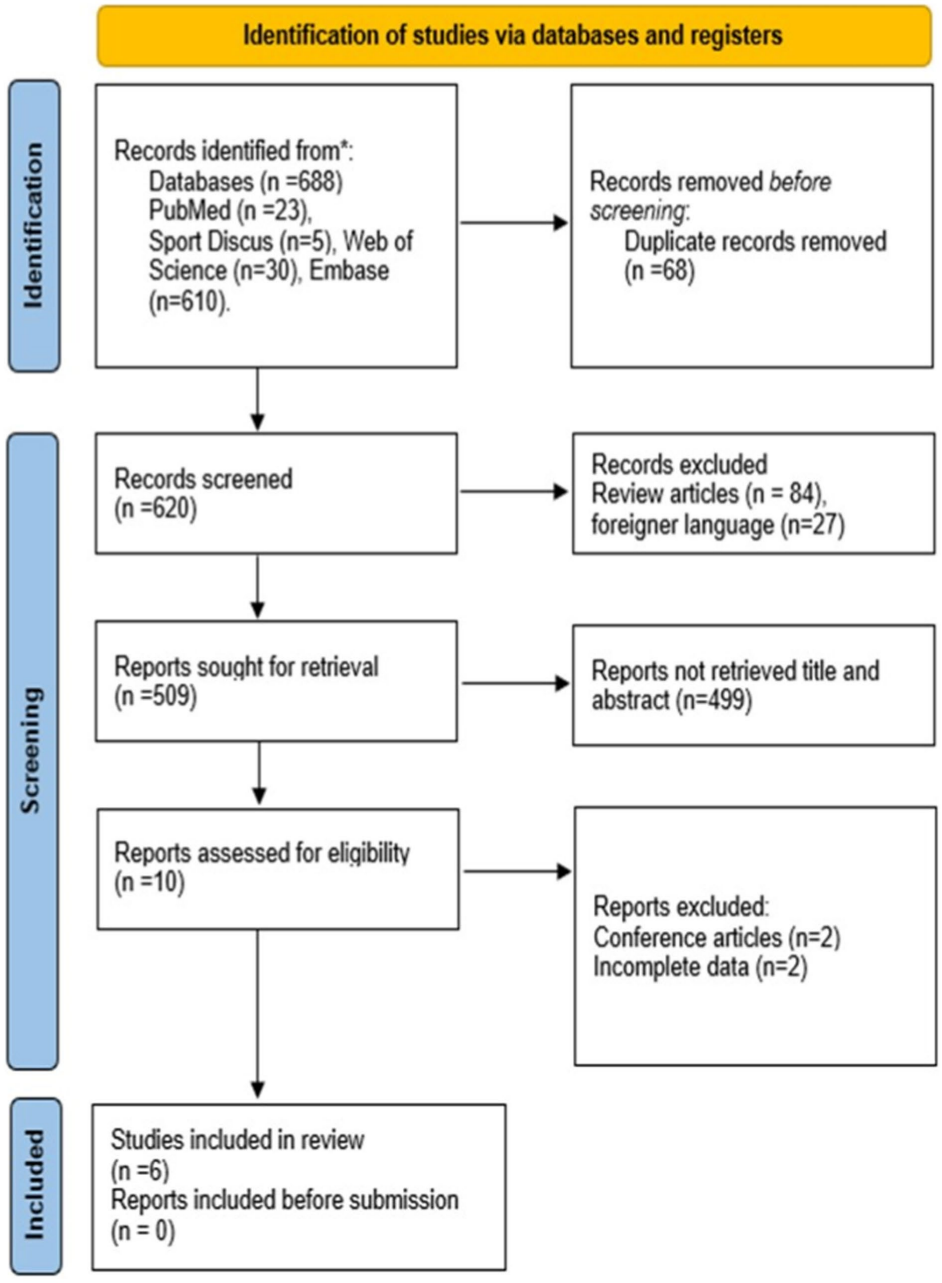
Flowchart diagram of the study selection process (PRISMA 2020).

Six hundred and eighty-eight records were found in database searching. After duplicate removal, we screened 620 records, from which 84 studies were excluded that presented a review method and 27 with a foreign language. Therefore, 509 was retained to screen title and abstract, of which 499 were excluded because they do not present a description of orthorexia scores in athletes. The last phase was to take 10 articles to read full text, and after excluding articles such as reports, no data description, and not using the questionnaire specific (ORTO-15), it was included in review 6 articles. Later, we search documents that cited any of the initially included studies as well as the references of the initial included studies. However, no extra articles that fulfilled inclusion criteria were found in these searches (Figure 1).

Table 1 presents the characteristics and outcomes of the articles included. The athletes from the articles selected in this review presented different competitive levels ranging from student athletes (Clifford and Blyth, 2019) to elite athletes (Surala et al., 2020), and athletes from specific sports modalities such as wheelchair basketball (Toti et al., 2021). The studies were conducted mostly in Italy (Segura-García et al., 2012; Bert et al., 2019; Toti et al., 2021) and evaluated athletes from both sexes (Segura-García et al., 2012; Bert et al., 2019; Clifford and Blyth, 2019; Surala et al., 2020; Uriegas et al., 2021).

**Table 1:** Characteristics of the included studies and relevant outcomes.

	Sample characteristics					Outcome athletes (cutoff = 40)	Comparison
Study	Country	Modality	Sex	Age (years)	Sample size (*n*)		Male vs. Female (athletes)
Surala et al. (2020)	Poland	19 Olympic sports	M/F	20.9 ± 4.7	273	35.3 ± 3.5 ↓*	M = 35.1 ± 3.1↓* F = 35.4 ± 3.7↓*
Uriegas et al. (2021)	United States	Athletes from different sports	M/F	19.6 ± 1.4	1,090	37.7 ± 3.7 ↓*	M = 38.2 ± 3.4↓* F = 37.5 ± 3.8 ↓*
							*Athletes* versus *No-Athletes*
Bert et al. (2019)	Italy	Athletes from different sports	M/F	26.7 ± 5.39	549	37.26 ± 3.75 ↓*	Control no sport: 37.59 ± 3.63↓*
Clifford and Blyth (2019)	UK	University athletes	M/F	21 ± 1	215	36.6 ± 3.9 ↓*	No-athletes = 37.2 ± 3.9↓*
Segura-García et al. (2012)	Italy	Athletes from different sports	M/F	16–45	794	M = 37.2 ± 4.3↓* F = 36.7 ± 4.2 ↓*	Control M = 37.7 ± 3.4 ↓* Control *F* = 38.4 ± 3.9 ↓*
Toti et al. (2021)	Italy	Wheelchair basketball	M	28.5 ± 1.5	60	36.9 ± 1.1 ↓*	34.7 ± 1.1 ↓

*Risk of orthorexia nervosa risk considering the cutoff = 35.

Considering the results from the athletes’ group described in each study, the six articles presented a cutoff below 40 points, demonstrating no risk of orthorexia nervosa according to the ORTO-15 interpretation (Donini et al., 2005). On the other hand, all studies presented results above 35 points, ranging the total scores from 38 to 35 points.

Comparison between male and female athletes was performed by two articles (Surala et al., 2020; Uriegas et al., 2021). In both articles, no significant difference was found between the orthorexic scores from males and females; also, the scores were below 40 points (and above 35 points). When comparing athletes with no-athletes, no significant difference also was found by the articles included.

## Discussion

The present review summarized the orthorexic profile in athletes and found that athletes from different disciplines presented scores ranging from 38 to 35 points of the ORTO-15 questionnaire. Therefore, the results demonstrated that the risk of orthorexia nervosa in athletes may vary depending on the cutoff point established. No difference in orthorexia scores was found between athletes and non-athletes, demonstrating that orthorexia is an issue that needs to be considered in the general population.

Regarding the cutoff point, the authors that developed the ORTO-15 questionnaire first considered predictive a score over 40 points to classify as the risk of presenting an orthorexic behavior (Donini et al., 2005). Nonetheless, previous studies have criticized the ORTO-15 and pointed out that a 35 cutoff point can maximize either sensitivity or specificity (Ramacciotti et al., 2011; Dell’Osso et al., 2016). In the present review, it is possible to notice that the risk of ON will depend on what cutoff will be chosen, highlighting the importance of establishing a better sensitivity to diagnose the prevalence of orthorexia, especially in athletes. As pointed out by Cena et al. (2019), it is still a challenge to *draw a boundary between adopting a healthy diet… and developing inflexible beliefs, attitudes, and behaviors related to nutrition with unhealthy consequences.*

The comparison of orthorexic profiles between sexes was explored recently, in a review article with the general population, showing similar tendencies toward healthy eating in men and women in the studies that used the ORTO-15 questionnaire (Strahler, 2019). Likewise, the studies included in this review also reported no significant differences between male and female athletes either in Olympics athletes (Surala et al., 2020) and professional (Uriegas et al., 2021) or students athletes (Clifford and Blyth, 2019). Furthermore, the athletes’ food behavior did not differ from the general population, showing an interesting and unexpected result.

In general, orthorexic behavior may manifest as dietary, behavioral, and exercise-related. Excessive training has been associated with an increased risk of low energy availability (Melin et al., 2019). Moreover, an association between unhealthy exercise behavior (e.g., exercise dependence or exercise addiction) and disordered eating has been found (Taranis et al., 2011). The distinction between athletes and non-athletes must be made in terms of highly variable exercise behavior. Athletes undertaking high training volume and low energy availability may occur without disordered eating due to unintentionally failing to consume sufficient energy (Loucks et al., 2011). In agreement with the present findings, interestingly a high prevalence of orthorexia symptoms *via* ORTO-15 was also found in the non-athletic healthy eating community on Instagram (Turner and Lefevre, 2017) and yoga practitioners (Herranz Valera et al., 2014; Domingues and Carmo, 2019, 2021). Altered eating behavior as a consequence of body dissatisfaction attributed to the pressure experienced by athletes through social media has also been proposed (Wasserfurth et al., 2020).

### Limitation and Further Directions

The ON is an emerging topic of investigation, especially in sport settings, and the present review brings new pieces of information about this field; however, there are some limitations to highlight. A small number of articles included as well as the different characteristics of athletes amongst the studies could be a limitation on the generalization of the results. Additionally, the review considered a cutoff of 40 and 35 points as a risk of orthorexia nervosa from ORTO-15; nonetheless, it is not a consensus yet about the standard diagnostic criteria or tool to diagnose the orthorexic behavior.

Therefore, special attention should be given to the interpretation of the results from the ORTO-15 questionnaire in order to identify the risk of ON. Questionnaires focused on disordered eating are crucial parts of screening tools of athletes at risk of RED-S (Sim and Burns, 2021), and identifying athletes at risk of orthorexia (e.g., involved leanness demanding sports) by adopting ORTO-15 as an additional tool to generate more information may increase the sensitivity of diagnosis of disordered eating.

## Conclusion

The results summarized in this review demonstrated that athletes from different sport modalities presented no risk of orthorexia nervosa considering the cutoff of 40 points but not for the cutoff of 35 points in the ORTO-15 questionnaire. The athletes’ scores ranged between 38 and 35 points; thus, attention should be given considering the diagnoses and classification about orthorexic behavior when using the ORTO-15 in athletes, to avoid false-negative results. The non-difference between athletes and non-athletes demonstrated that orthorexia nervosa is a concern of the general population. However, these assumptions should be given using ORTO-15 as a complementary tool to diagnose orthorexia nervosa or any other disordered eating may be recommended to avoid the biased result. This review adds a piece of information about the orthorexia nervosa and ORTO-15 questionnaire topic, recommending further studies to improve the quality of psychometric instruments to facilitate the use in non-clinical populations.

## Author Contributions

AP, MM, HM, MVM, and MK provided article conceptualization. AP and MM were involved in search and data analysis. AP, MM, HM, and MK drafted the manuscript. AP, MM, HM, MVM, and MK critically revised the manuscript. All authors contributed to the article and approved the submitted version.

## Funding

This project was supported by the Evaluation of Graduate Education, and from Operational Programme Research, Development and Education–Project “Postdoc2MUNI” (No.CZ.02.2.69/0.0/0.0/18_053/0016952).

## Conflict of Interest

The authors declare that the research was conducted in the absence of any commercial or financial relationships that could be construed as a potential conflict of interest.

## Publisher’s Note

All claims expressed in this article are solely those of the authors and do not necessarily represent those of their affiliated organizations, or those of the publisher, the editors and the reviewers. Any product that may be evaluated in this article, or claim that may be made by its manufacturer, is not guaranteed or endorsed by the publisher.
